# Beyond the Gates: Identifying and Managing Offenders with Attention Deficit Hyperactivity Disorder in Community Probation Services

**DOI:** 10.3934/publichealth.2014.1.33

**Published:** 2014-03-17

**Authors:** Susan Young, Gisli H Gudjonsson, Emily J Goodwin, Amit Jotangia, Romana Farooq, David Haddrick, Marios Adamou

**Affiliations:** 1 Centre for Mental Health, Division of Brain Sciences, Department of Medicine, Charing Cross Campus, Imperial College London, Claybrook Centre, 37 Claybrook Road, London W6 8LN, UK; 2 Broadmoor Hospital, Crowthorne, UK; 3 Institute of Psychiatry, King's College London, UK; 4 North London Forensic Service, Barnet, Enfield & Haringey Mental Health Trust, UK.; 5 Manygates Clinic, South West Yorkshire Partnership NHS Foundation Trust, UK; 6 West Yorkshire Probation Trust, UK

**Keywords:** ADHD, offenders, prevalence, probation, services, community, rehabilitation, crime, neuropsychology

## Abstract

Research has indicated that, compared with the general population, the prevalence of offenders with ADHD in prison is high. The situation for offenders managed in the community by the Probation Service is unknown. This study aimed to bridge the gap in our knowledge by (1) surveying the awareness of probation staff about ADHD and (2) screening the rate of offenders with ADHD managed within the service. In the first study, a brief survey was circulated to offender managers working in 7 Probation Trusts in England and Wales asking them to estimate the prevalence of offenders with ADHD on their caseload, the presenting problems of these offenders and challenges to their management, and the training received on the treatment and management of offenders with ADHD. The survey had a return rate of 11%. Probation staff perceived that 7.6% of their caseload had ADHD and identified this group to have difficulties associated with neuropsychological dysfunction, lifestyle problems and compliance problems. They perceived that these problems hindered meaningful engagement with the service and rehabilitation. Challenges to their management were perceived to be due to both internal processes (motivation and engagement) and external processes (inadequate or inappropriate interventions). Few respondents had received training in the management of offenders with ADHD and most wanted more support. In the second study, a sub-sample of 88 offenders in one Probation Trust completed questionnaires to screen for DSM-IV ADHD in childhood and current symptoms. The screen found an estimated prevalence of 45.45% and 20.51% for childhood and adulthood ADHD respectively and these were strongly associated with functional impairment. Thus probation staff considerably underestimated the likely rate, suggesting there are high rates of under-detection and/or misdiagnosis among offenders with ADHD in their service. The results indicate that screening provisions are needed in probation settings, together with training for staff.

## 1. Introduction

In recent years there has been growing acknowledgement of the high rates of young people with ADHD who come into contact with the criminal justice system. Rates vary depending on the screening methods and diagnostic criteria used, but a general consensus from data reported in international studies suggests that around 30% of adult male offenders in the prison population have ADHD [1]. A rate of 23.5% has been reported for those in police custody [2]. Youth offender rates may be higher [3],[4] and female adult rates may be lower [5]. This compares with general population rates of around 5% in children and 2.5% in adults [6],[7]. These young people with ADHD are reported to present in the criminal justice system at a younger age, even as young as 10 years old [8],[9]. They are four to five times more likely to be arrested and are more likely to have multiple arrests and convictions than those without ADHD [8]–[11]. They have greater clinical and personality pathology than their non-ADHD peers [12]–[14]. In custody they are more likely to present with demanding and/or aggressive behaviours [2],[15],[16].

Whilst the evidence base is growing for those detained in custodial settings, little is known about what happens ‘beyond the gates’ when many move into the care of probation services in the community. The role of criminal justice services in supporting offenders with ADHD in England and Wales was discussed at a meeting of experts from health (including representatives from the Department of Health) and criminal justice agencies in 2009. It was concluded that ADHD has begun to be recognized by the courts, prison and police services. However, a clear gap was identified within the probation service, which is a key service for providing support and management in the offender pathway. We therefore aimed to bridge this gap in knowledge by (1) surveying the awareness of probation staff about ADHD and (2) screening the rate of probation service-users with ADHD. In study 1 we investigated awareness by circulating a brief survey to offender managers working in seven Probation Trusts in England and Wales. In study 2 we estimated the actual prevalence rate of ADHD and associated functional impairment among a sub-sample of offenders managed within one Probation Trust using a screening protocol. It was hypothesized that rates of ADHD would be higher than those reported in the general population and similar to those reported in other forensic settings (H1); and that those screening positive for ADHD would report higher rates of impairment both in childhood and in their current functioning (H2).

## 2. Materials and Method

### 2.1. Study 1: Awareness about ADHD among Probation Staff

#### 2.1.1. Participants

All probation officers/offender managers (around 600) working across seven Probation Trusts in England and Wales were invited to complete the survey.

#### 2.1.2. Measures

A survey was designed to meet the specific aims of this study that asked offender managers to estimate the prevalence of offenders with ADHD on their caseload, comment on the presenting problems of these offenders and challenges to their management, and describe the training they had received on the treatment and management of offenders with ADHD (survey available from corresponding author).

#### 2.1.3. Procedure

Agreement to conduct study 1 was obtained from senior management in the Probation Trusts and ethical approval was awarded by the Psychiatry, Nursing and Midwifery RESC committee, reference PNM-1112-12. Senior management within each Trust circulated an email to all staff inviting them to participate and attaching an Information Sheet describing the purpose of the survey and providing a link to an online version. In addition a hard copy was attached to the email for staff who preferred to download the survey and return it by post.

### 2.2. Study 2: Estimated Prevalence of ADHD in the Probation Service

#### 2.2.1. Participants

A total of 108 male offenders managed in the community by the West Yorkshire Probation Trust and aged between 18 and 25 years participated in the study. The West Yorkshire Probation Trust is the fourth largest in England and Wales supervising around 12,000 cases at any one time, most of whom are in the community. The majority of service users are young men, thus females and/or those aged 26 or above were excluded from the study.

#### 2.2.2. Measures

Participants completed the Barkley Current and Childhood Symptoms Scales [17] to rate current (i.e. in the past six months) and childhood ADHD symptoms. These 18-item self-report questionnaires are based upon DSM-IV criteria (9 in each domain of attention and hyperactivity/impulsivity), and use a 4-point rating scale to indicate frequency of symptoms. ADHD in childhood was classified as present if an individual rated “often” or “very often” (i) six or more symptoms in either domain on the retrospective childhood rating scale. Current ADHD (i.e. in adulthood) was classified as present for those meeting criteria (i) plus (ii) six or more ADHD symptoms from either domain from current ADHD symptom rating scales.

The Barkley Symptoms Scales also include an assessment of impairment that enquires about function in specific areas of life activities. Participants rate the frequency with which they have experienced functional problems over the previous six months on a four-point scale for ten domains including home life, work or occupation, social interactions, community activities, educational activities, dating or marital relationships, money management, driving, leisure or recreational activities and management of daily responsibilities. Each domain can be summed to generate a total impairment score that ranges between 0 and 30.

In addition, the current probation status of the offender was recorded for categories of (1) offender in the community, (2) offender in approved premises/hostel and (3) persistent ‘prolific’ offender. The categories broadly represent low to high risk levels for re-offending and/or risk of harm towards others

#### 2.2.3. Procedure

Agreement to conduct study 2 was obtained by senior management in the West Yorkshire Probation Trust and approved by the Probation Research Board for England and Wales. Following a brief session of training in their administration, the ADHD rating scales (both child and current) were administered by staff to all offenders on their caseload who met inclusion criteria and who attended a routine healthcare assessment over a 12 month period. Probation staff read items to participants with literacy problems but they were given no further assistance or direction to complete the questionnaires.

## 3. Results

### 3.1. Survey Response Rate

The survey response rate was poor at around 11% (N=68 respondents).

### 3.2. Probation Staff's Estimate of ADHD Prevalence within Their Service

The average number of offenders per caseload was 37.7 (range = 2 to 113). Out of a total of 2,563 offenders on their caseloads, staff estimated that 206 had ADHD (7.6%).

### 3.3. Probation Staff's Perception of the Presenting Problems of Offenders with ADHD

Responses to the open question “Describe the three main presenting problems for ADHD offenders” indicated three main groups of difficulties, the most common relating to neuropsychological dysfunction (e.g. inattention, hyperactivity, impulsivity, memory, emotion regulation, planning, and organisation). These deficits were perceived to hinder meaningful engagement in rehabilitation (i.e. in group work, discussion) and attenuate progress, for example due to time-management problems, missed appointments, lack of prioritisation and/or comprehension problems.

The second category of presenting problems related to social and interpersonal problems (e.g. difficulties with relationships, social problem-solving, antisocial attitudes and behaviour, chaotic lifestyle, education and employment, substance misuse, health, and housing problems). Within this category probation staff described how core symptoms of ADHD can negatively influence social skills and interpersonal relationships, and suggested that ADHD offenders may be “pushed away” by positive role models and easily re-engage with criminal peers.

The third category related to difficulties associated with adhering to rehabilitation plans and included problems with attendance, maintaining appropriate boundaries, and accepting instructions. Low levels of self-esteem and motivation were also indicated, which may have hindered progress in their rehabilitation.

### 3.4. Probation Staff's Perception of Challenges to Managing Offenders with ADHD

Responses to the open question “Describe the three main difficulties in managing ADHD offenders” indicated two dominant categories relating to (1) internal processes (motivation and engagement), and (2) external processes (inadequate or inappropriate interventions). Motivational and engagement problems were perceived to be the primary management difficulty. One respondent mentioned how persisting, challenging behaviours often lead to the abandonment of motivational techniques, and teaching offenders with ADHD constructive life skills was perceived to be challenging due to their difficulties with self-regulation and limitations in social perspective taking.

External factors referred to concerns about the adequacy of treatment programmes for offenders with ADHD. They were perceived as not receiving appropriate medication, leading to them being difficult to manage both in prison and in the community. In particular, difficulties were experienced “keeping ADHD offenders on target”, with staff suggesting that high levels of tolerance were required for their successful management and progress.

### 3.5. Training

Around one-fifth (19.1%) had attended talks or presentations on ADHD and a small percentage (5.1%) had received some ADHD training. Almost three-quarters (73.5%) of respondents reported they did not have adequate support from mental health services to manage offenders with ADHD, and just over half (52.9%) believed that there should be specialist workers within the probation service for managing offenders with ADHD.

### 3.6. Screening Rates of ADHD within One Probation Trust

Of the 108 screens received, 20 were incomplete and could not be used. Of the remaining 88 participants, 53 (60.2%) were managed in the community, 17 (19.3%) were in a probation hostel, and 18 (20.5%) were classified as high risk or persistent prolific offenders.

Of this group, 40 (45.45%) screened positive for childhood ADHD, of whom 8 (20.00%) were of predominantly hyperactive/impulsive type, 7 (17.50%) predominantly inattentive type, and 25 (62.50%) combined type (see Table 1). Ten participants refused to complete the current symptom screen for adulthood ADHD (5 of whom met criteria for ADHD on the child screen). Of the 40 individuals who had screened positive on the child screen and completed the adult screen, 16 (20.51%) met criteria for ADHD in adulthood, of whom 5 (31.25%) were of predominantly hyperactive/impulsive type, 2 (12.50%) inattentive, and 9 (56.25%) combined type. Overall the data suggests an estimated child and adult prevalence of 45.45% and 20.51% respectively.

**Table 1.** Screening rates of ADHD in childhood and adulthood.

	Childhood screen n (%) (N=88)	Adulthood screen n (%)(N=78)
*No ADHD*	*48 (54.55%)*	*62 (79.49%)*
*ADHD*	*40 (45.45%)*	*16 (20.51%)*
Combined	25 (62.50%)	9 (56.25%)
Hyperactive/impulsive	8 (20.00%)	5 (31.25%)
Inattentive	7 (17.50%)	2 (12.50%)

### 3.7. Functional Impairment

As the impairment total variables did not meet criteria for parametric testing, Mann-Whitney U tests were conducted to compare the total impairment scores obtained on the child and adult ADHD symptom scales. A positive screen in childhood was associated with greater childhood (U=138.50, p < 0.001, r = 0.64) and current impairment (U = 82.50, p < 0.001, r = 0.64), both with large effect sizes and both remaining significant when a Bonferroni correction was made to account for the two analyses (see Table 2).

A similar pattern was found for current ADHD criteria which was associated with greater childhood (U = 91.00, p < 0.001, r = 0.58) and current impairment (U = 48.00, p < 0.001, r = 0.61). Again, both results showed large effect sizes and remained significant following the Bonferroni correction.

**Table 2.** Impairment scores comparing those who screened positive or negative for childhood and adulthood ADHD

Childhood screening results				
	*ADHD*	*No ADHD*	*Mann-Whitney U*	*Effect size (r)*
Childhood impairment median	12.00	3.00	138.50*	0.64
Current impairment median	9.00	1.00	82.50*	0.64

Adulthood screening results				
	*ADHD*	*No ADHD*	*Mann-Whitney U*	*Effect size (r)*
Childhood impairment median	18.00	4.50	91.00	0.58
Current impairment median	12.00	2.00	48.00	0.61

## 4. Discussion

This study is the first of its kind with a focus on the probation service and, as hypothesised, there was a significantly greater estimated rate of offenders with ADHD being managed in the community by probation services than reported in the general population. The rates obtained were generally consistent with those reported in prison studies e.g. [4],[5],[14],[18] with an estimated prevalence of 45.45% and 20.51% for childhood and adulthood respectively. Furthermore these offenders reported significantly greater functional impairment in both childhood and adulthood with large effect sizes, which has important implications regarding their ability to cope effectively in the community. The probation staff, by contrast, estimated that 7.6% of their caseload consisted of offenders with ADHD, suggesting that there may be high rates of under-detection and/or misdiagnosis among offenders on probation.

The offender managers identified that this group of offenders were more impaired than their non-ADHD peers and perceived that this impairment hampered their ability to engage with the service and successful progress in their rehabilitation. Particular issues related to neuropsychological, lifestyle and compliance problems and, for some, risk may be inflated due to these barriers that hamper their rehabilitation process. The casework relationship between offender managers and the offenders they work with involves a dual relationship of care and control yet they seemed to feel ill-equipped to support the offenders with ADHD on their caseload, identifying a clear training need. Very few offender managers reported they had received any training in ADHD and most responded that they needed more support from mental health services. Many reported that staff with specialist training in this area would be useful. Given the likely proportion that one-quarter of their caseload has ADHD, specialist supervision may be a cost effective solution.

The pharmacological treatment of offenders with ADHD may have profound effects for both patient care and societal gains. Lichtenstein and colleagues [19] gathered information on 25,656 patients with a diagnosis of ADHD, drawing on data in Swedish national registers recording pharmacological treatment and subsequent criminal convictions over a 3-year period. They found that 37% of males and 15% of females had been convicted of at least one crime. Importantly it was found that the use of ADHD medication reduced the crime rate by 32% and 41% for males and females respectively. Similar analyses were conducted for treatment with antidepressant or SSRI medications with no effect and it was concluded that among patients with ADHD, rates of criminality were lower during periods when they were receiving ADHD medication.

Another Swedish study conducted a randomised controlled trial investigating both symptom and functional outcomes of treating offenders with ADHD with stimulant medication in the prison setting [20],[21]. They reported a large treatment effect for both symptomatic and functional improvement (in both neuropsychological and quality of life domains) that was sustained over a 12-month period of study. No substance misuse was detected during the course of the study and the majority of participants took part in accredited treatment programmes and educational activities. Hence treatment with medication may not solely confer health gain to the individual but may also facilitate engagement with the service and the rehabilitation process. A treatment approach that combines pharmacological and psychological approaches, as recommended by international guidelines [22],[23], may be the most efficacious in meeting the needs of a complex client group who have high rates of comorbidity. A specific programme has been developed to meet the needs of patients with ADHD and antisocial behaviour, the R&R2 ADHD [24]. This programme is an adaptation of the internationally accredited Reasoning & Rehabilitation programme and a meta-analysis of outcomes obtained from 16 evaluations found that, compared with controls, recidivism was reduced by 14% and 21% when the programme was delivered in institutional and community settings respectively [25]. The R&R2 ADHD revision has been associated with large and sustained treatment effects for clinical improvement in a randomised community trial conducted in a community sample [26] and has been successfully piloted in offenders with personality disorder [27].

A strength of the current study is that it is the first one (so far as we know) to report the rates and management needs of offenders with ADHD in probation services. This is an important topic as the recognition and management of these offenders has the potential to benefit both the individual and society. The survey was conducted in a cross-section of the probation service that spanned a broad geographical area across the UK but there was a very low return rate. Nevertheless, those participating in the screening study were representative of offenders presenting with a broad spectrum of risk of re-offending and risk of harm towards others. However, the screening protocol was administered in only one Probation Trust and twenty screens were incomplete and had to be excluded from the sample. The Probation Trusts did not systematically record the number of offenders approached for inclusion in the study; the sample size was small and only male which may have further limited generalisability. In common with many prevalence studies of ADHD that apply a screening protocol, this study used self-reported rating scales to screen for symptoms of ADHD, one of which asks the participant to retrospectively recall symptoms in childhood which may not be accurate [28]. Whilst the sensitivity and specificity of such ratings scales is unknown and a diagnosis should only follow a comprehensive clinical interview [22],[29], they may nevertheless be useful for indicating whether a more detailed clinical assessment is warranted.

## 5. Conclusion

There is a consistent over-representation of offenders with ADHD presenting in custodial and community probation settings. With respect to the latter, this study has highlighted two important needs. Firstly, screening protocols and procedures are required in probation settings in order to assist offender managers to identify offenders who may be affected by persisting symptoms of ADHD. Unidentified symptoms are likely to impact on both the ability of the offender to use the support that is available and the ability of staff to tailor appropriate treatment and support. Secondly there is a clear training need to support staff in their management of a challenging group of offenders with a high rate of functional impairment who may have difficulty adhering to a community treatment protocol and who are likely to benefit from treatment. The evidence regarding the vulnerabilities of offenders with ADHD in the criminal justice system is growing. We now have the knowledge base and something needs to be done as the evidence is taking us beyond ‘food for thought’ and towards a call for action.
